# National survey of perinatal professionals’ experiences and approaches to conducting prebirth periviable (22+0 to 23+6 weeks) conversations: a mixed-methods study

**DOI:** 10.1136/bmjpo-2025-004153

**Published:** 2026-10-28

**Authors:** Jennifer L H Peterson, Kate Delany, Edward D Johnstone, Ajit Mahaveer, Debbie M Smith

**Affiliations:** 1 The University of Manchester Faculty of Biology Medicine and Health, Manchester, UK; 2 Manchester University NHS Foundation Trust, Manchester, UK

**Keywords:** Neonatology, Intensive Care Units, Neonatal, Infant

## Abstract

**Background:**

Conducting prebirth conversations with parents facing periviable birth (22+0 to 23+6 weeks) presents numerous challenges for perinatal professionals. This mixed-methods survey aimed to gather a holistic assessment of the experiences and issues encountered by UK-based perinatal professionals in relation to periviable birth.

**Methods:**

A voluntary, anonymous national online survey of perinatal professionals was conducted using QualtricsXM, developed with input from a parental advisory group. The survey was structured into discrete sections exploring different elements of prebirth conversations and incorporated vignette-based scenarios (22+3, 23+3, 24+3 weeks) to explore professionals’ experiences of prebirth periviable discussions, including communication, moral distress and training needs. It was distributed through relevant professional organisations across the UK between March and April 2022. Quantitative data were summarised descriptively and qualitative free-text responses were analysed using reflexive thematic analysis.

**Results:**

The survey received 387 views with responses across the perinatal multidisciplinary team (MDT). The completed response rate varied from 100 respondents (questions related to training) to 321 (clinical vignette questions).

In contrast to national guidance, the survey showed that only two-thirds of paediatric and neonatal consultants and one-third of obstetric consultants would include comfort care within prebirth discussions with parents. Additionally, the survey demonstrated that involvement in these conversations confers a moral distress burden to perinatal professionals across the MDT.

**Conclusion:**

Variation in management options presented to parents has the potential to limit their ability to be meaningfully involved in decisions relating to their baby. Further research is required to determine how best to train and support perinatal professionals to deliver this challenging area of care.

**WHAT IS ALREADY KNOWN ON THIS TOPIC**

- Survival and neurodevelopmental outcomes are improving for infants born at 22+0 to 23+6 weeks. However, there remains significant uncertainty around how to optimise care.

**WHAT THIS STUDY ADDS**

- This UK-based survey is the first to explore challenges perinatal professionals face during prebirth periviable counselling.
- Compared with national guidelines, results show variability in professionals’ discussion of the important topic of comfort care with parents.
- Involvement in these conversations confers a burden of moral distress to professionals, often managed through peer discussion and reflection.

**HOW THIS STUDY MIGHT AFFECT RESEARCH, PRACTICE OR POLICY**

- The survey provides evidence that professionals desire experiential, multidisciplinary training, including simulation training and feedback from specialist centres and parents, to improve their periviable counselling practices.

## Introduction

 Survival and neurodevelopmental outcomes are continually improving for periviable infants.^1^ The periviable period refers to the timepoint in pregnancy where the infant may be able to survive outside the womb with the provision of intensive care. The exact gestational age where this occurs depends, in part, on the healthcare system, facilities and geographical location of the infant. In the UK in 2025, this would be between 22+0 and 23+6 weeks gestation.^2^ For infants born in this periviable period, it can no longer be considered futile to provide survival-focused care as survival outcomes have increased to between 30% and 80% internationally.^2 3^ However, at present, perinatal professionals lack the tools to determine before birth which infants will be able to survive and which will be the ones for whom the burden of intensive care and associated complications are deemed too great, and comfort care may have been the more appropriate option.^4^ Providing intensive care to infants born in the periviable period involves multiple invasive procedures and interventions potentially associated with pain, and even the process of providing basic care to the infant can cause iatrogenic harm, for example, skin injuries through application of vital monitoring equipment.^5^

Due to variable survival outcomes and risks of severe neurodevelopmental complications, there remain significant levels of uncertainty about the optimal management for these infants and this places decisions regarding management at birth (survival-focused or comfort care) within the zone of parental discretion, where professionals should integrate the parental view and perspective into the overall decision for the baby. Decision-making in these circumstances carries a high risk of perinatal professionals experiencing conflict between their own moral values and the course of action determined by the parent/s and other professionals within the perinatal multidisciplinary team (MDT). This internal conflict is termed moral distress.^6^ It can arise in situations where there is uncertainty about prognosis and an absence of evidence to help guide management. These factors make involvement in periviable birth high risk for professionals to experience moral distress. As described by Prentice *et al*, the ‘anguish associated with moral distress is legitimate and real. It originates from a sincere concern that the current management plan is not in the patient’s interests’.^7^ It is crucial that perinatal professionals can differentiate between distress caused by the professional desire to protect the baby, the drive to avoid being the agent causing iatrogenic harm to the baby and the moral distress caused through having to make decisions in circumstances of significant uncertainty, in order to accurately address the root cause of the distress and recognise that the presence of moral distress does not necessarily equate with the determined course of action (or management) being inappropriate. Moral distress is a complex and intensely uncomfortable experience for the individual and is linked to increased rates of stress, burnout, fatigue, depression and post-traumatic stress disorders in professionals.^8^

## Aim

Given all these complexities, this survey aims to gather a holistic assessment of the experiences of perinatal professionals working in the UK and the issues encountered when conducting prebirth periviable conversations with parents.

## Methods

### Patient and public involvement

The research team met with parents who had experienced periviable birth through the local parental advisory group (PAG) prior to designing the survey. The PAG consisted of parents with lived experience of extreme preterm and periviable birth with a range of outcomes. Parents recognised that periviable birth presented significant practical and ethical challenges for perinatal professionals and that management practices and outcomes for these infants were constantly evolving. They recognised the pressures that perinatal professionals were under when managing a periviable birth. Parents described multiple instances where communication from perinatal professionals needed to be improved. These included examples of parents being told different, conflicting gestational age range limits for when intensive care would be offered by different members of the perinatal team. Parents reported these changing messages were distressing and created a feeling of distrust.

Based on this feedback, the survey includes questions built around vignettes of periviable infants at three different periviable gestations (22+3, 23+3 and 24+3 weeks) with the aim of eliciting the topics that perinatal professionals would include in prebirth discussions with parents, including that of the complex topic of comfort care, which may be difficult but necessary to discuss within the periviable birth context.

### Survey design

The survey was developed and reported in accordance with the Checklist for Reporting Results of Internet E-Surveys (CHERRIES)^9 10^ and the Consolidated criteria for Reporting Qualitative research (COREQ) checklist for qualitative components.^11^

An online survey was selected to enable national distribution across perinatal professionals and provide ease of engagement for respondents. The survey was built by the research team using QualtricsXM.^12^ This platform was selected as it enabled researchers to develop a structured, cohesive-appearing survey, allowing ease of navigation and completion for respondents and ensuring secure data entry and storage (QualtricsXM approved by Information Governance for the University of Manchester^12 13^). This was a voluntary online survey of perinatal professionals with no financial or motivational incentives to participate. Therefore, as per the National Health Service Health Research Authority online assessment,^14^ formal ethics approval was not required.

Respondents were informed of the scope of the survey, who was conducting the research and the estimated length of time for completion on the introductory page of the survey. A copy of the survey can be viewed in online supplemental appendix A. The survey required respondents to enter their perinatal profession. No identifiable information was collected. The survey did not collect data about their geographical location. The questions were not mandatory and respondents could navigate through the survey freely and they did not have to complete the survey for their responses to be counted.

The survey was designed to explore different elements of professionals’ experiences of the prebirth periviable conversation. The survey was structured into discrete sections to address these different elements, asking questions related to:

1. Personal role, experience and reactions to these conversations.
  - This section included specific questions about whether professionals had experienced moral distress in relation to these conversations.
  - Definitions of moral distress and how it differs from emotional distress were provided (survey provided in full in online supplemental appendix A).
    - ‘Emotional distress refers to any emotions you may experience around these conversations. Moral distress refers to any situations where you may experience internal conflict between your own moral values and having to providing care that may not completely align with these’*.*
2. Topics included in these conversations.
3. Professionals’ experience of receiving training for these conversations and ideas for future training modalities.

### Survey distribution

The survey was an open survey, distributed through relevant professional organisations across the UK. The aim was to capture responses from across the perinatal MDT who would be regularly involved in prebirth periviable conversations. Therefore, the survey was distributed through the mailing lists of the British Association of Perinatal Medicine (BAPM), Royal College of Obstetricians and Gynaecologists, Royal College of Midwives and UK-wide RCPCH-listed paediatric training programme directors. The survey was live between 1 March and 29 April 2022. Results were analysed by professional group. All responses were anonymous.

### Survey analysis

The survey aimed to gather a wide range of perinatal professionals’ experiences over a range of topics. It was anticipated that not all respondents would complete the entire survey. Data were gathered on the number of views of the survey and the number of responses per question. All submitted responses were analysed. Results were subdivided by professional group. Given differing response rates between different professional groups, quantitative results have been presented with percentage comparisons across professional groups. Qualitative responses were analysed by JLHP and KD using Braun and Clarke’s six-step reflexive thematic analysis.^15^ The free-text responses regarding moral distress experiences were collated and reviewed independently by JLHP (neonatal doctor) and KD (medical student). Both JLHP and KD received training in conducting reflexive thematic analysis and were supervised by DMS, who is a perinatal researcher with extensive experience conducting qualitative research, including thematic analysis. Following data familiarisation and initial independent theme identification by JLHP and KD, the final set of themes was agreed through an iterative joint discussion process. A third author (DMS) cross-checked the final set of themes against the raw data to ensure themes had been directly derived from the data.

Distribution of the survey was through multidisciplinary professional channels, resulting in the survey being distributed to a wide scope of perinatal professionals, not all of whom would be involved in prebirth conversations. For example, the BAPM mailing list will contain numerous perinatal professionals, including allied health professionals, such as speech and language therapists, who are unlikely to be involved in prebirth conversations. Therefore, the denominator selected for this survey was to compare the number of responses received against the number of views of the survey. This would represent the proportion of professionals who determined the survey relevant to themselves (through clicking the link to the survey), who then went on to submit responses to the survey.

## Results

The survey received responses within each section from across the perinatal MDT. The survey was viewed by 387 respondents. There was a variable response rate for different sections of the survey. The personal experience and moral distress section received 171 responses (44% completion rate). The clinical content of prebirth conversations section received 321 responses (83% completion rate). The final training section received the lowest response rate, with between 100 and 185 respondents (26%–48% completion rate) depending on the specific question.

### Moral distress burden for perinatal professionals

The survey data show that moral distress has been experienced universally across the perinatal professions involved in prebirth periviable counselling (figure 1). The extent of the moral distress experienced varied by professional group, with the highest burdens being within the paediatric and neonatal teams.

**Figure 1:**
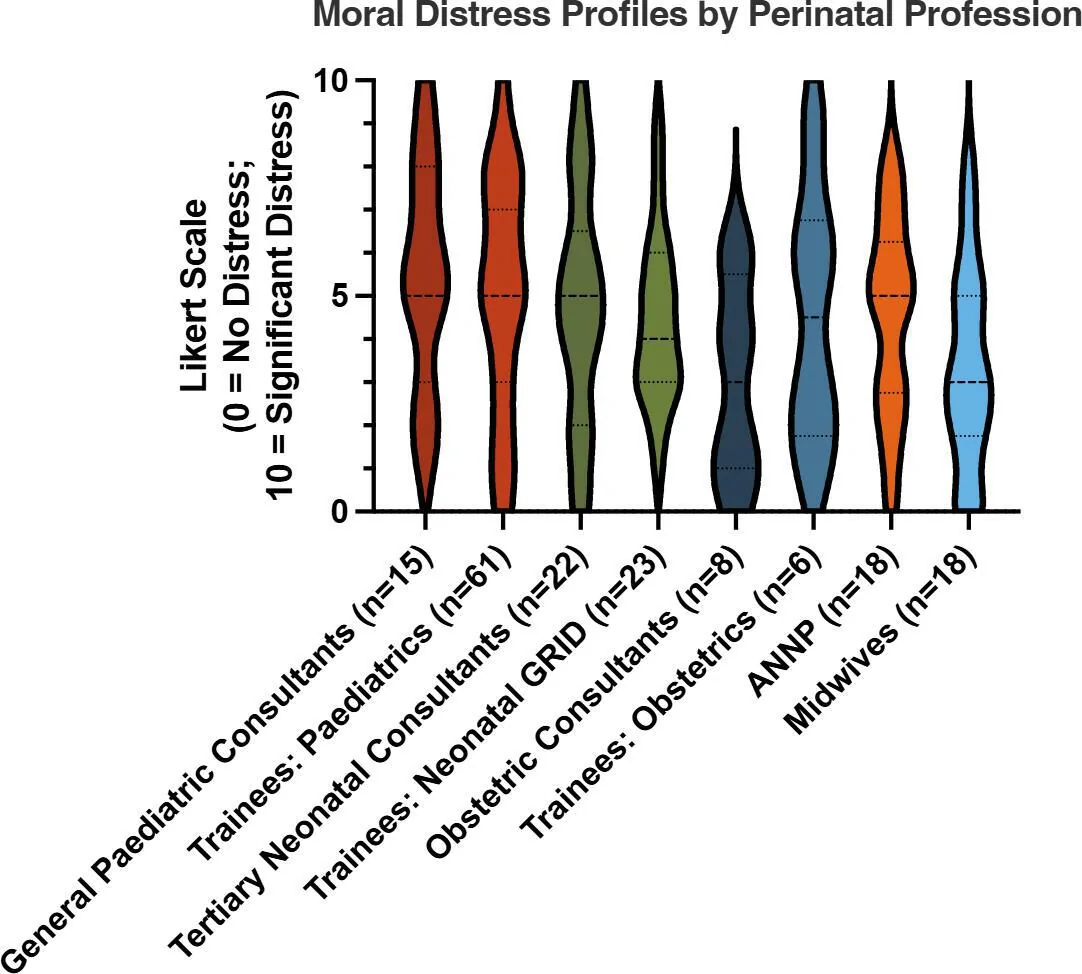
Moral distress profiles by perinatal profession. ANNP, advanced neonatal nurse practitioner.

Professionals who had experienced moral distress were asked to provide a free-text answer about which element/s of the prebirth discussions evoked that distress. The survey received 38 free-text responses to this question. From these responses, six key domains were identified. Moral distress occurred predominantly through the professional perception that the decision being made was not in the ‘baby’s best interests’ (14 responses (37%)) and concerns that parents were not able to fully comprehend the risks of death and long-term complications for them and their baby (eight responses (21%)). Other professionals reported that the uncertainty that existed within these situations caused them moral distress (seven responses (18%), including one respondent who stated their moral distress occurred through ‘asking parents to agree to comfort care’. Additionally, two respondents described their distress arising in situations of perceived professional dishonesty, which they self-reported as discussing survival-focused care with expectant parents in a non-specialist centre while knowing that outcomes for infants are significantly worse if delivered outside a specialist centre.

The survey data showed varied strategies for how perinatal professionals managed their moral distress (figure 2). From the 171 respondents to this question, there were 183 statements regarding strategies used for managing moral distress. The main strategies across all professional groups were:

1. Discussion with colleagues (exemplar quote: “I process these thoughts verbally within the safety of my team. They are able to hold the complexity of the situation and my processing”).
2. Professional compartmentalisation or detachment (exemplar quote: “There is a useful time to compartmentalise and accept that this is what we do. I think we do what we can do”).
3. Self-reflection (exemplar quote: “I find it difficult to watch patients suffer because parents are desperate for them to survive but I can appreciate that hope prevails”).

**Figure 2:**
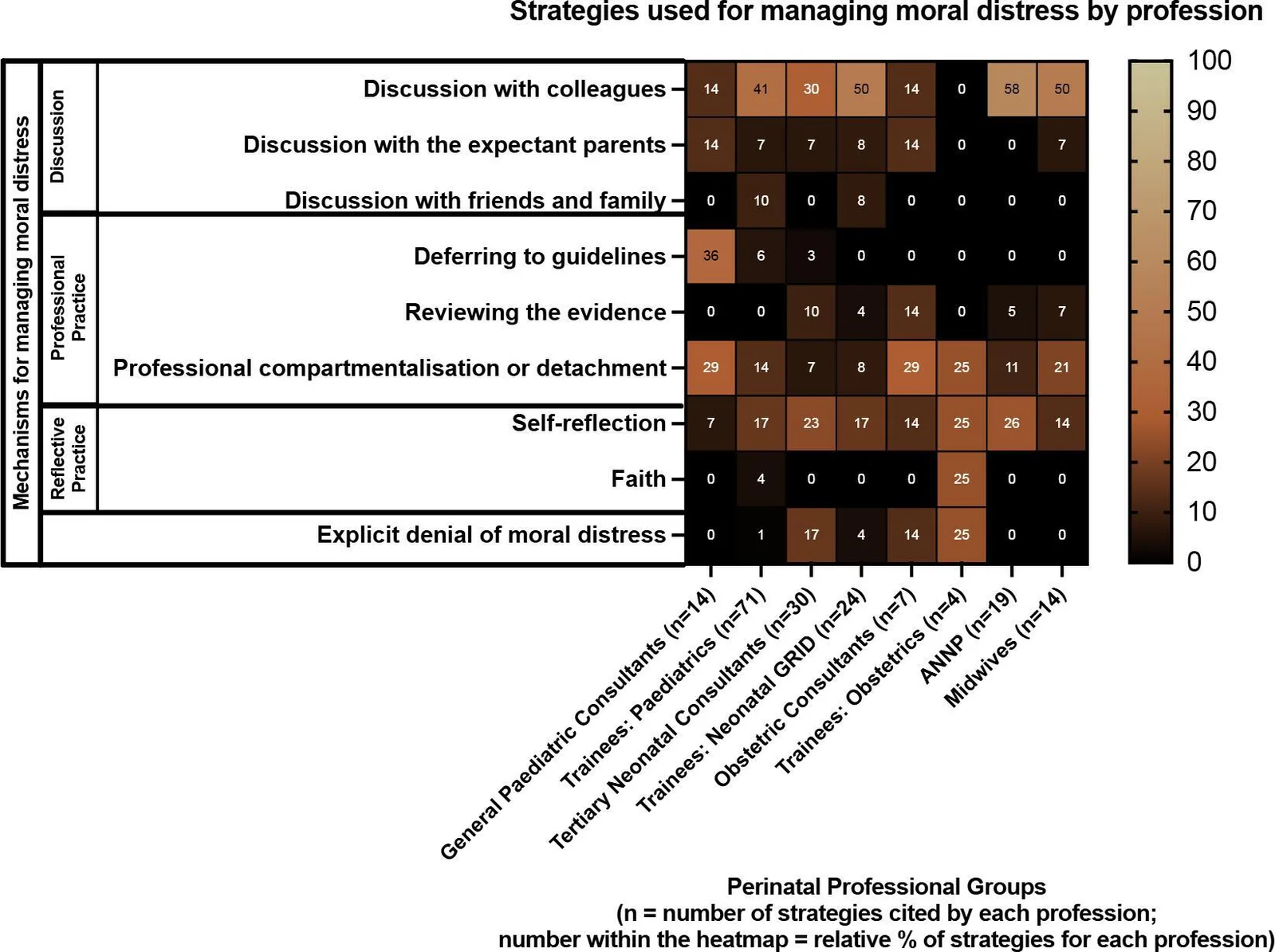
Strategies used for managing moral distress by profession. ANNP, advanced neonatal nurse practitioner.

Strategies of discussing with colleagues and friends or family were reported more frequently among paediatric, neonatal and midwifery professionals, and less commonly among obstetricians. Small proportions of perinatal professionals across the MDT reported finding it helpful to have had detailed discussions with the expectant parents themselves. These statements centred around the professional checking that the parent/s ‘are as fully and realistically informed as they can be regarding their options’. Others referenced turning towards their faith to aid them with their moral distress burden; ‘I pray’.

Additionally, as seen within figure 2, some professionals explicitly denied experiencing any moral distress over these conversations. These included statements such as, ‘It [moral distress] does not exist’ and ‘I don’t have any moral distress. I believe that nature didn’t intend for baby to survive; nature is cruel’. These statements of denying the experience of moral distress were more prevalent in the obstetric and tertiary neonatal consultant professional groups.

### Content of periviable conversations with parents

This section of the survey focused on the content of the prebirth conversation. The three vignettes provided involved the professional attending a consultation with parents in early labour at (22+3/23+3/24+3) weeks with a female infant with an average estimated weight for gestation and there is time for antenatal steroids and magnesium sulphate. Professionals were then requested to outline the topics they would include in the prebirth conversations at each of the provided periviable gestational ages.

#### Discussion of comfort care

There were 321 responses to this set of questions with responses from across the perinatal MDT. The vignettes featuring infants at 22+3 and 23+3 weeks would fall within the ‘high’ risk category of the BAPM risk stratification framework where discussion of both survival-focused and comfort care with parents would be the recommended approach. For the vignette featuring the 24+3-week infant the BAPM risk stratification framework would classify the infant as ‘moderate’ risk and would recommend a survival-focused approach.

The survey results demonstrate markedly lower rates of including comfort care in the prebirth conversation at 22 and 23 weeks gestation than would be expected (figure 3). The advanced neonatal nurse practitioner (ANNP) group was the only group who would include discussion of comfort care within all prebirth conversations with parents. Even within the ANNP cohort, this decreased to 67% of ANNPs including comfort care within prebirth conversations for the 23+3-week vignette, despite this vignette also falling into the ‘high’ risk category. For the other professional groups, inclusion of comfort care within the prebirth conversation was varied. In response to the 22+3-week vignette, discussion of comfort care within prebirth conversations was included by approximately two-thirds of paediatric and neonatal consultants (16–30 respondents (57–64%)) and only one-third of obstetric consultants (5 respondents (29%)) and midwives (12 respondents (33%)). Given that the professional framework would recommend comfort care being discussed with parents in all cases for the 22+3 and 23+3-week vignettes, the data show that inclusion of comfort care rates within the survey cohort were much lower than expected.

**Figure 3:**
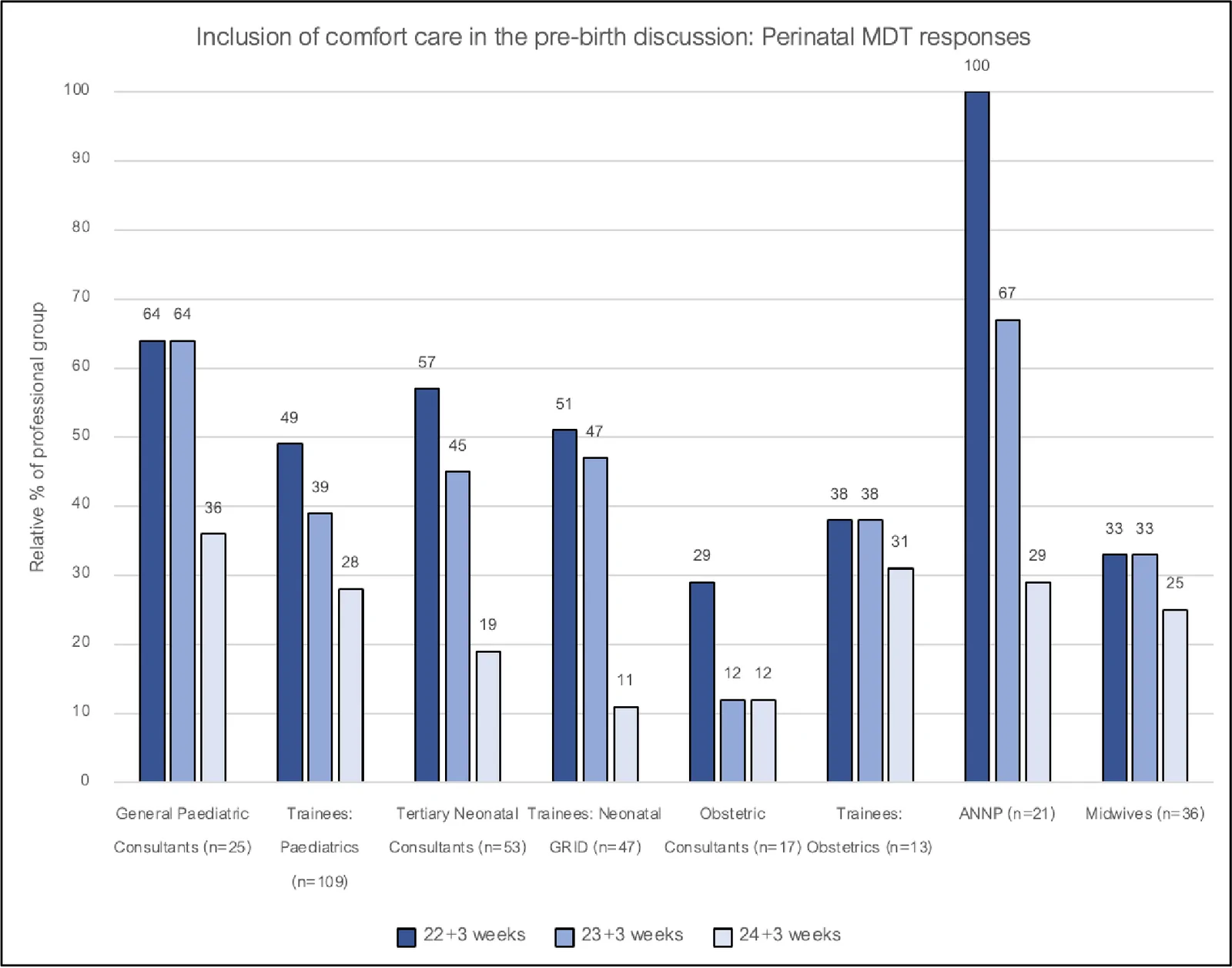
Inclusion of comfort care within the prebirth conversation with parents. ANNP, advanced neonatal nurse practitioner; MDT, multidisciplinary team.

### Perinatal professionals’ experiences of training for these prebirth conversations

The final section of the survey looked at education and training for perinatal professionals involved in prebirth periviable conversations. Professionals were asked about if they had received specific training for these types of conversations. There were 100 respondents to this question from across the perinatal MDT and a range of training types were reported (figure 4a). Some respondents reported receiving training via more than one method. Most respondents (60%) considered that they had been trained by observing senior colleagues conducting these conversations and learning from them. Simulation training, which includes simulation-based courses such as the Advanced Resuscitation of the Newborn Infant course, was reported by 30% of respondents. Other training methods reported included lecture-based, didactic teaching about guidelines related to periviable infants (15%) and attending formal communication skills courses (5%).

**Figure 4:**
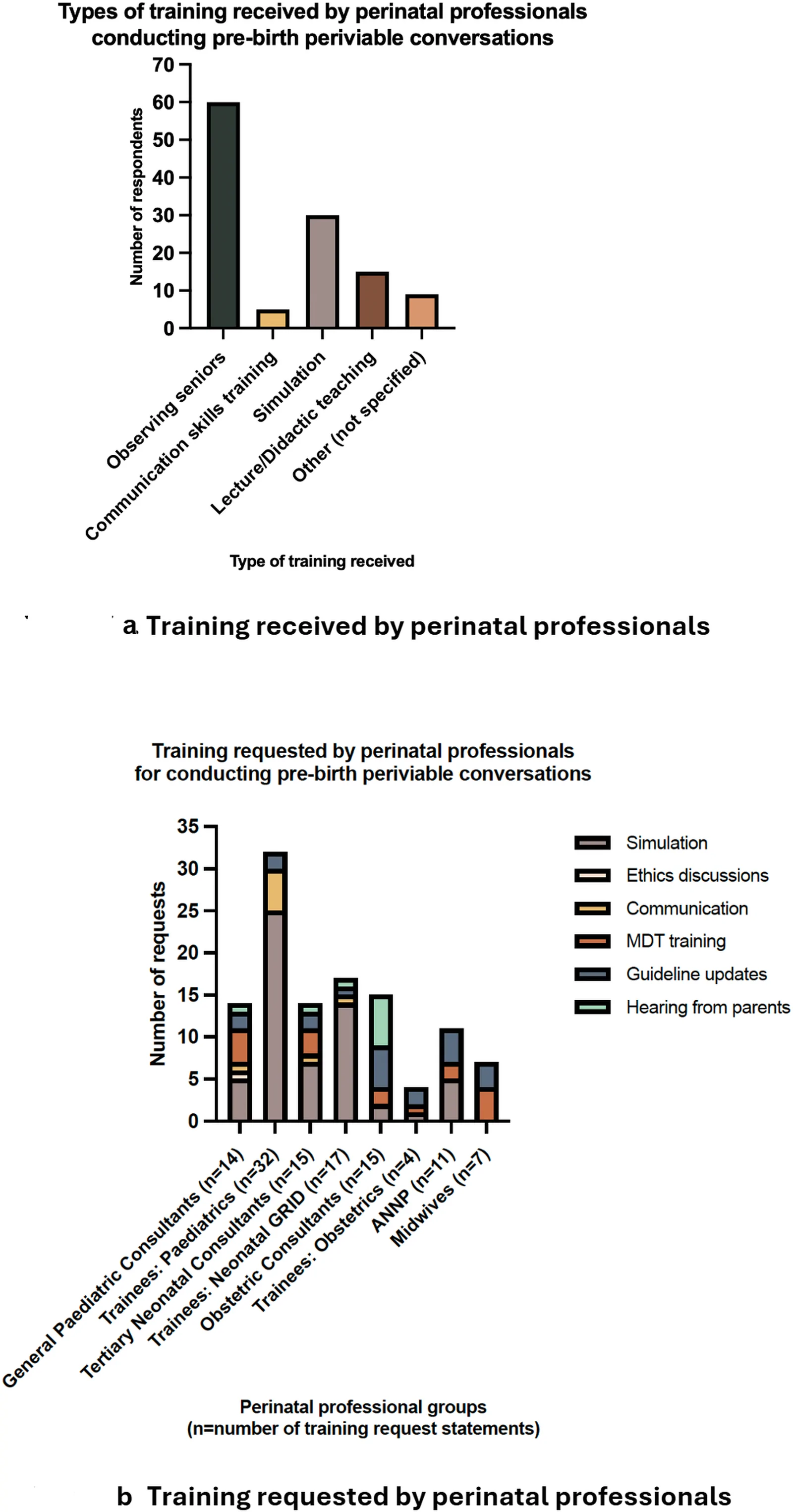
(a) Training received and (b) training requested by perinatal professionals. ANNP, advanced neonatal nurse practitioner; MDT, multidisciplinary team.

Perinatal professionals were then asked about the type of training they would like to receive in relation to prebirth periviable conversations. This question received 115 responses from across the perinatal MDT (figure 4b). Simulation was the most requested training modality (59 respondents (51%)), with emphasis in these statements for the simulation focusing on communication with parents. Respondents also requested guideline updates on evolving practice in relation to this group of infants (21 respondents (18%)) and for specific joint training and data sharing sessions with input from specialist centres, such as the tertiary neonatal team (16 respondents (14%)). There were nine respondents (8%) who stated they would like to hear from parents with lived experience of periviable birth.

## Discussion

This survey has gathered perspectives from across the perinatal MDT regarding numerous components and considerations within the complex issue of the prebirth periviable conversation. The survey results showed that while professionals reported discomfort and moral distress from involvement in these conversations, there was a simultaneous, unexpected reluctance to discuss the option of comfort care. This discordance of discomforts, both with being asked to provide intensive care and also with discussing comfort care, reflects the moral and communication complexities of this conversation.

### Comfort care

The survey demonstrates that while professionals are aware of national frameworks available to help guide these conversations and decision-making processes, there remains variation in which management options and outcomes professionals deem appropriate to include in discussions with parents. The survey shows that even in scenarios where the periviable infant would be considered by professional frameworks to be ‘high’ risk and therefore both comfort care and survival-focused care options should be discussed with the parents,^2^ that this was not the case. Discussing comfort care and the dying process with parents can be emotive and challenging for professionals. There may be concerns that talking about comfort care could appear as if the team have ‘given up’ on the baby and professionals may worry that these conversations will be distressing for the parents.^16^ However, omission of this option denies parents an opportunity to consider the full range of options available, reducing their autonomy and diminishing their role as parents within the decision-making process. Additionally, given the high-risk nature of periviable deliveries, even in cases where survival-focused care is deemed most appropriate, there remains risk of death for these infants. Therefore, it is important for perinatal professionals to integrate parallel planning and palliative care practices within their discussions, right from the prebirth conversations onwards.^16^ Through acknowledging and discussing the uncertainties periviable birth raises, the potential outcomes for the baby, eliciting the parents’ perspectives on these issues and facilitating parents to explore their views on different potential journeys their periviable baby may face, integration of discussions of comfort care can be sensitively and appropriately navigated. Paternalism from perinatal professionals should not prevent parents from being able to access information about the full range of relevant management options available to them. For infants delivered at 22–23 weeks, as per professional frameworks, these options should include discussion of comfort care.^2^ Research into information sharing practices between professionals and parents facing periviable birth show that transparency of information, including professionals not withholding relevant information, is crucial to establishing trust and enabling parental involvement in decisions about their baby.^17–19^ Evidence shows lower rates of decisional regret when parents feel they have been actively involved in decision-making processes.^20^ Additionally, the experience of periviable birth can lead parents to experience trauma through a loss of control over their own circumstances, body and what is happening to their baby.^19^ Professionals can help reduce this trauma by supporting parents through ‘good parent belief’ principles.^21^ These principles, derived from research from parents of critically ill or dying children, have demonstrated the desire from parents to be able to fulfil their parental role to their child and that this can be enabled through parents being fully informed about issues relating to their child’s care, being able to alleviate their child’s pain and being able to ensure their child feels loved.^22^ Naturally, different parents will respond to these principles in different ways at different timepoints. However, professionals have a responsibility to ensure that all parents are informed about all relevant management options and to be empowered to make loving, considered decisions for their baby. In the context of 22- and 23-week periviable infants, this should include a discussion of comfort care within the prebirth conversation.

### Moral distress

This survey is the first evaluation of the experience of moral distress for perinatal professionals working in the UK in relation to the issue of periviability. The survey data show that involvement in prebirth periviable conversations can cause moral distress for professionals and that this was present across the perinatal MDT. Moral distress has been associated with increased rates of burnout, depression, post-traumatic stress disorders and an increased likelihood of leaving the profession.^8^ It is important for perinatal professionals to recognise the presence of moral distress within this area of perinatal care and to be able to reflect on which elements of periviable care are contributing to that distress. Cognitive errors here can lead to professionals holding unbalanced ethical positions on the issue of periviability; this was observed in some of the survey responses reporting their moral distress originated from providing survival-focused care to 22-week infants as this was ‘futile’ and ‘against the infant’s best interests’. Given the evolving survival rates for 22-week infants, intensive care can no longer be considered futile.^3^ The uncertainty experienced by professionals in relation to periviable management is present and difficult to navigate but the presence of uncertainty means professionals cannot be certain that provision of survival-focused care is against the infant’s best interests: the alternative here is certain death if comfort care is implemented, making assessment of ‘best interests’ decidedly problematic.

The survey shows that perinatal professionals used a range of strategies to manage their moral distress. Of note, the strategies most reported were discussion with colleagues and self-reflection. This aligns with wider research on narrative medicine programmes which show benefits for professionals participating in reflective practice sessions which focus on non-clinical aspects of medicine, such as uncertainty, and can be beneficial in the management of moral distress.^23–25^ Narrative medicine is an educational and practical approach to the daily practice of clinical medicine which aims to use art, reading and reflective writing to improve the professional’s competence with skills of communication, empathy and reflection.^26 27^ The survey showed a clear desire from perinatal professionals for training in conducting prebirth periviable conversations with parents and for these sessions to be interprofessional across the whole perinatal team. Multidisciplinary training within perinatal medicine has been a recommendation from large-scale maternity reviews, such as the recent Ockenden review which outlined that ‘staff that work together should train together’.^28^ Based on the survey results, there appear to be distinct roles for development of multidisciplinary simulation training and narrative medicine reflective practice sessions to optimise perinatal professional training and practice in relation to managing moral distress burdens and improving how prebirth periviable conversations are conducted.

### Strength and limitations

This survey is the first evaluation of the issues perinatal professionals working in the UK face in relation to conducting prebirth periviable conversations with parents. This survey invited participation from perinatal professionals from across the country and across the perinatal MDT. The survey is novel in that it included questions specifically evaluating the presence of moral distress in relation to prebirth periviable conversations and the coping strategies used by perinatal professionals in the UK.

The survey faced several limitations, including determining the denominator for calculating the survey response rate. The survey received variable response rates depending on the specific question within the survey. This may indicate that professionals felt certain questions were more or less applicable to their role or may indicate that the survey was too broad in its scope and/or had too many questions to hold respondents’ interest over the entirety of the survey. Professionals can receive numerous survey requests from professional societies and survey fatigue is a recognised phenomenon and may have influenced response rates. Additionally, the survey was distributed through professional society emails to members, with no guarantee how many individual professionals will read these emails. Due to this, it also became problematic to select a denominator for the survey response rate, as the professional mailing lists would include a plethora of roles within that profession, not all of whom would be directly involved in prebirth periviable conversations. For instance, the Royal College of Midwives report having 50 000 members,^29^ not all of whom will be practising in roles which would necessitate attending to women and their partners experiencing periviable birth. While the survey did gather responses from 321 perinatal professionals, the lack of a discrete denominator limits the confidence in the generalisability of the findings.

## Conclusion

*“This is one of the most complex discussions one could have as a doctor.*” (Survey respondent)

Conducting prebirth conversations with parents facing periviable birth is challenging for perinatal professionals. This survey has shown that involvement in these conversations is a source of moral distress for professionals across the perinatal MDT. Variation exists between delivery room management options (such as comfort care) deemed appropriate by perinatal professionals for discussion with parents and those recommended by national professional frameworks. Parents need access to accurate, transparent information relevant to their specific circumstances. This is necessary to ensure parents are given opportunities to be meaningfully involved in decisions relating to their baby’s care and to establish trust between professionals and parents.

This survey highlights that perinatal professionals receive variable training in conducting these nuanced prebirth conversations, with most professionals being informally trained through observing senior colleagues. Future medical education research should focus on the development of multidisciplinary simulated communication scenarios relating to periviable birth. These results also highlight that further research is required to explore, understand and improve the integration of narrative medicine and reflective practice sessions within perinatal professional training, aiming to equip professionals to be able to acknowledge and reflect on their experiences with uncertainty and moral distress.

## Supplementary material

10.1136/bmjpo-2025-004153 online supplemental file 1

## Data Availability

All data relevant to the study are included in the article or uploaded as supplementary information.
