# Supplementary material for: National survey of perinatal professionals’ experiences and approaches to conducting prebirth periviable (22+0 to 23+6 weeks) conversations: a mixed-methods study
Source: BMJ Paediatr Open. 2026 Oct 28;10(1):e004153. doi: 10.1136/bmjpo-2025-004153 (PMC13723893; doi:10.1136/bmjpo-2025-004153)
Supplement: Supplementary file 1 [file bmjpo-10-1-s001.pdf]

For the purpose of this survey, the term periviable relates to deliveries at 22+0 - 24+6 weeks gestation.

Default Question Block

This survey aims to gather Consultant perspectives on experiences and priorities during pre-delivery conversations with parents presenting in periviable (22+0 - 24+6 weeks) labour.
There are 4 sections to this survey. There are no right or wrong answers so please be honest and give as much detail as possible. Answers are anonymous.
This survey is likely to take 10 - 15 minutes to complete.
We appreciate you taking the time to complete this survey.

Block 1

What specialty are you?

- ○ Obstetrics
- ○ General Paediatrics
- ○ Tertiary Neonates

Have you ever attended periviable pre-delivery counselling (22+0 - 24+6 weeks)?

- Yes
- No

What is the youngest periviable gestation pre-delivery counselling you have attended?

Block 6

Section One: This section relates to your personal reactions to being involved in pre-delivery periviable conversations.
Emotional distress refers to any emotions you may experience around these conversations.
Moral distress refers to any situations where you may experience internal conflict between your own moral values and having to providing care that may not completely align with these.

Block 2

Does attending these periviable pre-delivery conversations cause you any emotional distress? (0 = No distress; 10 = Significant distress)

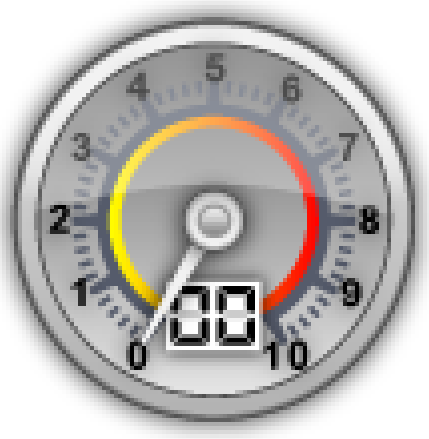

In what way have these conversations caused you emotional distress?

Does attending periviable pre-delivery conversations cause you any moral distress? (0 = No distress; 10 = Significant distress)

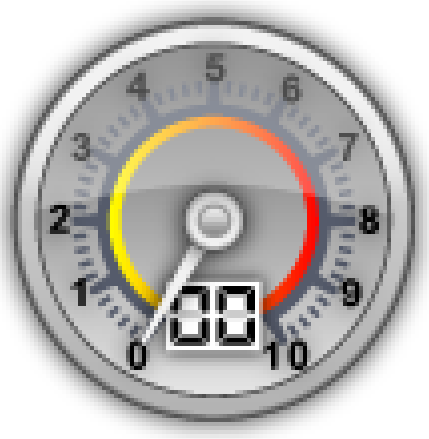

In what way have these conversations caused you moral distress?

How do you deal with this moral distress?

Block 7

Section Two: This section relates your experiences of the structure and content of pre-delivery periviable conversations.

Block 3

For the periviable pre-delivery conversations you have attended, who was present for the conversation?

[ ]

Where have you conducted periviable (22+0 - 24+6 weeks) pre-delivery conversations?

- ☐ Maternity Unit with a Level 1 Neonatal Unit (SCBU)
- ☐ Maternity Unit with a Level 2 Neonatal Unit (LNU)
- ☐ Maternity Unit with a Level 3 Neonatal Unit (NICU)
- ☐ N/A

When did the conversation take place? (Tick as many as apply)

- ☐ In labour: early stages
- ☐ In labour: late stages
- ☐ In labour: imminently about to deliver
- ☐ Not in labour: in hospital for monitoring
- ☐ Not in labour: in clinic
- ☐ [ ] Other

Have you been involved in any cases where there have been more than one pre-delivery conversation between the medical team and the same family during their periviable labour?

- ○ Yes
- ○ No
- ○ [ ] Other

Based on your previous experiences, if you were attending a consultation with parents in early labour at **24+3 weeks** with a female infant with an average estimated weight for gestation and time for antenatal steroids and magnesium sulphate, then what topics would you anticipate discussing with the parents? (Tick as many as apply)

- ☐ Delivery room basic airway support
- ☐ Delivery room intubation
- ☐ Delivery room surfactant
- ☐ Delivery room chest compressions
- ☐ Delivery room emergency drugs
- ☐ Comfort care at delivery
- ☐ Trial of intensive care with potential to reorientate to end of life care on NICU if not responding or significant complications

- ☐ Neonatal unit: ventilation
- ☐ Neonatal unit: feeding
- ☐ Neonatal unit: parental presence and Family Integrated Care (FIC) practices eg parent feeding, positive touch
- ☐ Neonatal unit complications: infection
- ☐ Neonatal unit complications: NEC
- ☐ Neonatal unit complications: IVH/cerebral palsy
- ☐ Neonatal unit complications: Chronic Lung Disease
- ☐ Neonatal unit complications: PDA
- ☐ Neonatal unit complications: pain or discomfort from multiple procedures
- ☐ Impact of trauma from neonatal intensive care on infant and/or parents and family
- ☐ Potential for there to be a positive impact on the family in raising a child born at periviability
- ☐ Potential for there to be a negative impact on the family in raising a child born at periviability
- ☐ Home oxygen
- ☐ Sensory processing issues
- ☐ Autism/ASD
- ☐ Additional learning needs
- ☐ [ ] Other

Based on your previous experiences, if you were attending a consultation with parents in early labour at **23+3 weeks** with a female infant with an average estimated weight for gestation and time for antenatal steroids and magnesium sulphate, then what topics

would you anticipate discussing with the parents? (Tick as many as apply)

- [ ] Delivery room basic airway support
- [ ] Delivery room intubation
- [ ] Delivery room surfactant
- [ ] Delivery room chest compressions
- [ ] Delivery room emergency drugs
- [ ] Comfort care at delivery
- [ ] Trial of intensive care with potential to reorientate to end of life care on NICU if not responding or significant complications
- [ ] Neonatal unit: ventilation
- [ ] Neonatal unit: feeding
- [ ] Neonatal unit: parental presence and Family Integrated Care (FIC) practices eg parent feeding, positive touch
- [ ] Neonatal unit complications: infection
- [ ] Neonatal unit complications: NEC
- [ ] Neonatal unit complications: IVH/cerebral palsy
- [ ] Neonatal unit complications: Chronic Lung Disease
- [ ] Neonatal unit complications: PDA
- [ ] Neonatal unit complications: pain or discomfort from multiple procedures
- [ ] Impact of trauma from neonatal intensive care on infant and/or parents and family
- [ ] Potential for there to be a positive impact on the family in raising a child born at periviability
- [ ] Potential for there to be a negative impact on the family in raising a child born at periviability
- [ ] Home oxygen
- [ ] Sensory processing issues
- [ ] Autism/ASD

- [ ] Additional learning needs
- [ ] [ ] Other

Based on your previous experiences, if you were attending a consultation with parents in early labour at **22+3 weeks** with a female infant with an average estimated weight for gestation and time for antenatal steroids and magnesium sulphate, then what topics would you anticipate discussing with the parents? (Tick as many as apply)

- [ ] Delivery room basic airway support
- [ ] Delivery room intubation
- [ ] Delivery room surfactant
- [ ] Delivery room chest compressions
- [ ] Delivery room emergency drugs
- [ ] Comfort care at delivery
- [ ] Trial of intensive care with potential to reorientate to end of life care on NICU if not responding or significant complications
- [ ] Neonatal unit: ventilation
- [ ] Neonatal unit: feeding
- [ ] Neonatal unit: parental presence and Family Integrated Care (FIC) practices eg parent feeding, positive touch
- [ ] Neonatal unit complications: infection
- [ ] Neonatal unit complications: NEC
- [ ] Neonatal unit complications: IVH/cerebral palsy
- [ ] Neonatal unit complications: Chronic Lung Disease
- [ ] Neonatal unit complications: PDA

- ☐ Neonatal unit complications: pain or discomfort from multiple procedures
- ☐ Impact of trauma from neonatal intensive care on infant and/or parents and family
- ☐ Potential for there to be a positive impact on the family in raising a child born at periviability
- ☐ Potential for there to be a negative impact on the family in raising a child born at periviability
- ☐ Home oxygen
- ☐ Sensory processing issues
- ☐ Autism/ASD
- ☐ Additional learning needs
- ☐ [ ] Other

Do you think medical staff should make recommendations in pre-delivery periviable conversations for how extensively to intervene at delivery?

- ○ Yes
- ○ No
- ○ [ ] Other

During the pre-delivery periviable (22+0 - 24+6 weeks) conversations you've been part of, was comfort care an option offered to the parents by the medical team? Or

did parents request comfort care? Was comfort care not mentioned?

If comfort care was discussed, what gestation was the pregnancy and what risk factors were present? (Leave blank if not applicable).

During periviable (22+0 - 24+6 weeks) pre-delivery conversations, are there any circumstances in which you would push for active, survival-focused care in situations where parental preference is for comfort care?

Block 8

Section Three: This section relates to your experience with existing guidelines and frameworks

relating to pre-delivery periviable conversations.

Block 4

Have you seen the BAPM Perinatal Management of Extreme Preterm Birth Before 27 weeks of Gestation Framework (2019) being used for periviable counselling?

- ○ Yes
- ○ No
- ○ [ ] Other

Did you find the Framework helpful or not? And why?

[ ]

Does your department have a local guideline for pre-delivery interventions and delivery management of periviable infants (22+0 - 24+6 weeks)?

- ○ Yes
- ○ No

○ [ ] Other

Have there been any barriers you have experienced to following this guideline (if applicable)?

[ ]

Block 9

Section Four: This section relates to your experience of staff training for pre-delivery periviable conversations and how training could be improved.

Block 5

Have you had any training in periviable pre-delivery counselling?

[ ]

If so, what training have you received?

What type of training/education would you like regarding pre-delivery periviable counselling, if any?

How do you evaluate whether the conversation was helpful for the parents?

For clinicians who have experience of multiple pre-delivery periviable conversations with parents, how do you feel your approach to these conversations has changed over time?

Powered by Qualtrics
