# Supplementary material for: National survey of perinatal professionals’ experiences and approaches to conducting prebirth periviable (22+0 to 23+6 weeks) conversations: a mixed-methods study
Source: BMJ Paediatr Open. 2026 Oct 28;10(1):e004153. doi: 10.1136/bmjpo-2025-004153 (PMC13723893; doi:10.1136/bmjpo-2025-004153)
Supplement: Supplementary file 2 [file bmjpo-2025-004153.reviewer_comments.pdf]

PEER REVIEW HISTORY

BMJ Paediatrics Open publishes all reviews undertaken for accepted manuscripts. Reviewers are asked to complete a checklist review form and are provided with free text boxes to elaborate on their assessment. These free text comments are reproduced below.

ARTICLE DETAILS

**TITLE (PROVISIONAL)**	A national survey of perinatal professionals' experiences and approaches to conducting pre-birth periviable (22+0-23+6 weeks) conversations
**AUTHORS**	Peterson, Jennifer<br>Delany, Kate<br>Johnstone, Edward<br>Mahaveer, Ajit<br>Smith, Debbie M

VERSION 1 – REVIEW

**REVIEWER NAME**	Jovandaric, Miljana
**REVIEWER AFFILIATION**	University Clinical Center of Serbia, Department of Neonatology
**REVIEWER CONFLICT OF INTEREST**	No Conflict of Interest
**AI DISCLOSURE**	No, I have not used AI
**DATE REVIEW RETURNED**	15-Jan-2026

**GENERAL COMMENTS**	good

**REVIEWER NAME**	Pillay, Thillagavathie
**REVIEWER AFFILIATION**	University Hospitals of Leicester NHS Trust, Neonatology
**REVIEWER CONFLICT OF INTEREST**	No Conflict of Interest
**AI DISCLOSURE**	No, I have not used AI
**DATE REVIEW RETURNED**	10-Mar-2026

**GENERAL COMMENTS**	Thank you for this important work. The area of reporting is topical and would be of interest in shaping the future of perinatal training for professionals.<br>Here are some areas to consider improving on:<br>a) A major limitation is sampling bias, reporter bias and recall bias in self reported data that is not triangulated by other means - how did the team consider that this could be balanced. Did the PAG recommend a parallel evaluation of parent perspectives.<br>b) The qualitative methodology is very limited and requires more detail. For example, how was data coded and themes derived, etc. Additionally, I could not see the CHERRIES checklist.<br>c) There are some areas of repetition and the article will benefit from being more concise.

VERSION 1 – AUTHOR RESPONSE

Reviewer 1 comment: Good.
Response: Thank you.

Reviewer 2 comments: Thank you for this important work. The area of reporting is topical and would be of interest in shaping the future of perinatal training for professionals.
Response: Thank you.

Reviewer 2 comments: A major limitation is sampling bias, reporter bias and recall bias in self reported data that is not triangulated by other means - how did the team consider that this could be balanced. Did the PAG recommend a parallel evaluation of parent perspectives.
Response: We agree that the survey format has the potential to introduce bias as you describe. We have aimed to be transparent in acknowledging this limitation and outline this in the methods and limitations sections.
The PAG did indeed support a parallel evaluation of parental perspectives in relation to this topic. This work has been conducted as a combination of a parent survey of parents with surviving children born at periviability (this manuscript is currently under review with BMJ Paediatrics Open) and also, a qualitative study where we conducted in-depth semi-structured interviews with parents and perinatal professionals about their experiences with the pre-birth conversation (https://www.frontiersin.org/journals/pediatrics/articles/10.3389/fped.2025.1552911/full)

Reviewer 2 comments: The qualitative methodology is very limited and requires more detail. For example, how was data coded and themes derived, etc. Additionally, I could not see the CHERRIES checklist.
Response: Agreed. We have expanded the methods section description of how the qualitative component of the survey was conducted. We have ensured that both the CHERRIES and COREQ checklists have been uploaded for your review.
